# *Toxoplasma gondii* dense granule protein 15 induces apoptosis in choriocarcinoma JEG-3 cells through endoplasmic reticulum stress

**DOI:** 10.1186/s13071-018-2835-3

**Published:** 2018-04-17

**Authors:** Wei Wei, Fangfang Zhang, He Chen, Yuanyuan Tang, Tian Xing, Qingli Luo, Li Yu, Jian Du, Jilong Shen, Linjie Zhang

**Affiliations:** 1 Department of Immunology, School of Basic Medicine, Anhui Medical University, Hefei, 230032 China; 2 Department of Pathogen Biology and the Key Laboratory of Microbiology (Anhui), School of Basic Medicine, Anhui Medical University, Hefei, 230032 China; 3 Laboratory of Clinical Diagnostics, the First Hospital of Anhui Medical University, Hefei, 230032 China; 4 Key Laboratory of Oral Diseases Research of Anhui Province, Hospital of Stomatology, Anhui Medical University, Hefei, 230032 China

**Keywords:** *Toxoplasma gondii*, GRA15, Apoptosis, ERS, JEG-3 cell

## Abstract

**Background:**

*Toxoplasma gondii*, a single-celled parasite commonly found in mammals, has been shown to induce trophoblast cell apoptosis and subsequently cause fetal damage and abortion. Although dense granule protein 15 (GRA15) has been identified as a key component in innate immunity to *T. gondii* infection and its pathogenesis, its role in host cell apoptosis remains unclarified.

**Methods:**

Type II GRA15 (GRA15_II_) cDNA was inserted into a plasmid encoding enhanced green fluorescent protein (pEGFP). Choriocarcinoma JEG-3 cells were transfected with either pEGFP or pEGFP-GRA15_II_ and cultured for 24 h. Cell apoptosis and endoplasmic reticulum stress (ERS) responses were assessed. Inhibitors targeting inositol-requiring kinase 1α (IRE1α; 4μ8C, 100 nM) or c-Jun N-terminal kinase (JNK; SP6000125, 20 μM) were added 12 h after plasmid transfection, followed by testing the effect of GRA15_II_ on ERS.

**Results:**

When compared to pEGFP, pEGFP-GRA15_II_ transfection facilitated cell apoptosis (*P* < 0.05), increased mRNA expression of caspase-3, caspase-4, 78-kDa glucose-regulated protein (GRP78), C/EBP homologous protein (CHOP) and X-box binding protein-1 (XBP1) (all *P* < 0.05), and promoted protein expression of cleaved caspase-3, cleaved poly(ADP-ribose) polymerase, Bax, CHOP, GRP78, phospho-JNK, and phospho-IRE1α (all *P* < 0.05). The 4μ8C and SP6000125 decreased apoptosis and protein expression of XBP1s, CHOP, TNF receptor-associated factor 2 (TRAF2), phosphorylated apoptosis signal-regulating kinase 1 (ASK1), cleaved caspase-3, phospho-JNK, and Bax (all *P* < 0.05) in pEGFP-GRA15_II_ transfected cells.

**Conclusions:**

*Toxoplasma* GRA15_II_ induced ERS and subsequently caused apoptosis of choriocarcinoma JEG-3 cells.

## Background

*Toxoplasma gondii* is an obligate intracellular single-celled parasite that can invade all warm-blooded animals worldwide [1]. The strains of *T. gondii* circulating in Europe and North America can be grouped into three distinct genotypes, strains of Type I, Type II and Type III, according to the population structure [2–5]. During invasion, proteins from parasite organelles such as rhoptry proteins (ROPs) and dense granule proteins (GRAs) are released into host cells and are able to cause significant host damage [6, 7]. The genotype/strain polymorphism of ROP16 and GRA15 have both been widely observed in the literature [8]. It has been reported that ROP16 from type I RH stain (ROP16_I_), but not from type II ME49 stain (ROP16_II_), could directly phosphorylate the signal transducer and activator of transcription STAT3 and STAT6, and subsequently polarize macrophages to an M2 phenotype. In addition, GRA15 from type II ME49 strain (GRA15_II_), but not from type I RH strain (GRA15_I_), could phosphorylate nuclear factor-kappa B (NF-κB), and subsequently drive macrophages to an M1 phenotype [9]. We have previously shown that both ROP16_I_ and GRA15_II_ were present in the majority of *T. gondii* Chinese 1 strains found in China [10–14].

*Toxoplasma gondii* can hijack host cell apoptotic machinery and promote either an anti- or pro-apoptotic program depending on the parasite virulence and load, as well as the host cell type [15]. In the literature, increased apoptosis following *Toxoplasma* infection has been observed in spleen cells [16], neuronal cells [17] and choriocarcinoma cells [18]. Previously, we found that endoplasmic reticulum stress (ERS) is involved in *T. gondii*-induced apoptosis [19, 20], and that ROPs could trigger ERS-mediated apoptosis [21, 22]. However, the effect of GRAs (e.g. GRA15) on host cell apoptosis remains unclear.

Importantly, maternal *Toxoplasma* infection may give rise to congenital transmission of the parasite to the fetus through the placenta [23–26] and/or *via* interfering with the immune tolerance on maternal-fetal interface. Our previous studies indicated that infection with TgCwh3 (a virulent strain of Chinese 1) induced apoptosis of trophoblast cells, and subsequently caused adverse pregnancy outcomes in mice [27]. Angeloni et al. [18] observed that ME49 (type II)-infected BeWo cells become more susceptible to apoptosis than RH (type I)-infected BeWo cells. In view of the M1 bias induced by GRA15_II_, we postulated that a GRA15_II_-induced NF-κB-dependent proinflammatory cytokine profile is more likely to cause cell apoptosis when compared to a ROP16_I_-induced STAT3/STAT6-dependent proinflammatory cytokines [18, 28]. Here, we demonstrated that GRA15_II_ increased apoptosis in choriocarcinoma JEG-3 cells partially mediated by ERS.

## Methods

### Cell culture

JEG-3 cells (human choriocarcinoma cell line, ATCC, lot number HTB-36) were cultured in a humidified incubator (37 °C and 5% CO_2_) in minimum essential medium (MEM; Gibco, Carlsbad, CA, USA), supplemented with sodium bicarbonate (1.5 g/l; Gibco), sodium pyruvate (0.11 g/l; Gibco), penicillin (100 U/ml; Sigma-Aldrich, St Louis, MO, USA), streptomycin (100 mg/ml; Sigma-Aldrich) and fetal bovine serum (10%; Gibco).

### Plasmid construction and transfection

A plasmid encoding enhanced green fluorescent protein-C2 (pEGFP-C2) was purchased from BD Biosciences (Franklin Lakes, NJ, USA). The open reading frame encoding T*g*GRA15_II_ (omitted signal peptide of 1500 bp; http://toxodb.org) cDNA was reconstituted by RT-PCR using total RNA isolated from TgCtwh3 tachyzoites. The pEGFP-GRA15_II_ plasmid was constructed by inserting T*g*GRA15_II_ cDNA into the pEGFP plasmid as previously described [29]. JEG-3 cells were plated in 96-well plates (Corning, Corning, NY, USA) at a density of 10^4^ cells/ml, cultured for 24 h, then transfected with either pEGFP or pEGFP-GRA15_II_ using Lipofectamine 3000 (Invitrogen, Carlsbad, CA, USA) according to the manufacturer’s instructions.

### The presence of GRA15_II_

The expression of green fluorescent protein (GFP) was recorded using fluorescent microscopy (Olympus BX60, Tokyo, Japan) 24 h after transfection. The presence of GRA15_II_ protein in either pEGFP- or pEGFP-GRA15_II_-transfected JEG-3 cells was determined by Western blotting (24 h after transfection). Untransfected JEG-3 cells served as a control.

### Cell viability and apoptosis

Twenty-four hours following transfection, JEG-3 cell viability was measured using the Cell Titer 96 Aqueous One Solution Cell Proliferation assay kit (MTS, Promega, Madison, WI, USA) according to the manufacturer’s instructions. A phycoerythrin-annexin V apoptosis detection kit (flow cytometry based assay, BD Biosciences, Franklin Lakes, NJ, USA) was used to determine cell apoptosis. Briefly, cells were washed twice using cold phosphate buffered saline (PBS) and suspended in binding buffer (100 μl). Phycoerythrin-annexin V (5 μl) and 7-aminoactinomycin D (7-AAD, 5 μl) were added to the suspension and incubated for 15 min at room temperature, followed by addition of binding buffer (400μl). A flow cytometry assay was conducted within 1 h using FACSVerse (BD Biosciences) with FCS Express 4.0. Annexin V^+^/7-AAD^-^ represented early apoptotic cells and annexin V^+^/7-AAD^+^ represented late apoptotic cells. Untransfected cells served as a negative control. Cells treated with staurosporine (STS, 1 μM, 6 h; Sigma-Aldrich) and/or tunicamycin (TM, 1 μM, 24 h; Sigma) served as positive controls. Cells were also treated with 4μ8C, the inositol-requiring kinase 1α (IRE1α) inhibitor (100 nM, added 12 h after transfection, for 12 h; Selleck, Houston, TX, USA) and/or SP6000125, the c-Jun N-terminal kinase (JNK) inhibitor (20 μM, added 12 h after transfection, for 12 h; Selleck) to demonstrate the effect of GRA15_II_ on ERS in JEG-3 cells.

### Real-time PCR

Cells were harvested 24 h after transfection. Total RNAs were extracted, and cDNA was synthesized using the Thermo Fisher Scientific RevertAid First Strand cDNA Synthesis Kit (lot number K1621, Thermo Fisher Scientific, San Diego, CA, USA). Quantitative real-time PCR was performed using the SYBR-Green kit (Takara, Tokyo, Japan) with the ABI7500 system (Applied Biosystems, Carlsbad, CA, USA). Primers listed in Table 1 were synthesized by Shenggong Biotechnology (Shanghai, China). Gene expression levels were normalized to glyceraldehyde-3-phosphate dehydrogenase (GAPDH) levels and data was analyzed using the 2^-ΔΔCt^ method.

**Table 1:** Sequences of oligonucleotide primers used for real-time PCR

Target	Forward primer (5'-3')	Reverse primer (5'-3')	Product size (bp)
Caspase 3	GACAGACAGTGGTGTTGATG	TGGATGAACCAGGAGCCATC	132
Caspase 8	AACCTGGTACATCCAGTCAC	AAAGTAGGCTGAGGCATCTG	150
Caspase 9	AAGGTTTGAGGACCTTCGAC	GACTGCAGGTCTTCAGAGTG	184
Caspase 4	GTGGAGAAGGACTTCATTGC	CTGGAAGCATGTGATGAGTTG	108
ATF4	CAGCTACCACCCATAACAAG	GTGTCCATCACCTGACAGTC	138
ATF6	CAGCTACCACCCATAACAAG	GTGTCCATCACCTGACAGTC	133
GRP78	GTCCTTCTATGAAGGAGAAG	GAATCTTCCAACACTTTCTGG	117
CHOP	TGCAAGAGGTCCTGTCTTCAG	GCACTGACTCCTCGGAAC	106
XBP1	AAGGCGCTGAGGAGGAAAC	GGTTCTCAACTACAAGGCC	178
GAPDH	CTTCATTGACCTCAACTACATGG	CTCGCTCCTGGAAGATGGTGAT	134

*Abbreviations*: *ATF* activating transcription factor, *GRP78* 78-kDa glucose-regulated protein, *CHOP* C/EBP homologous protein, *XBP1* X-box binding protein-1, *GAPDH* glyceraldehyde-3-phosphate dehydrogenase

### Western blotting

Cells were harvested 24 h after transfection, washed with cold PBS, and lysed on ice using lysis buffer [50 mM Tris-HCl pH 7.4, 150 mM NaCl, 1% Triton X-100, 1% sodium deoxycholate, and 0.1% sodium dodecyl sulfate (SDS), supplemented with protease inhibitors cocktail (1%) and 1 mM phenylmethanesulfonyl fluoride]. The whole cell lysate was centrifuged (12,000× *g*, 10 min, 4 °C), and the supernatant was collected. Protein content was analyzed using a bicinchoninic acid (BCA) assay kit (Beyotime, Shanghai, China). Protein (20 μg) was electrophoresed on 10% SDS-polyacrylamide gels, and transferred to nitrocellulose membranes (Millipore, Billerica, MA, USA). The membranes were blocked using 5% fat-free milk powder (in PBS) and incubated with primary antibody (1:1000 dilution) for 12 h at 4 °C. Horseradish peroxidase-conjugated secondary antibody (1:5000 dilution) was then applied at room temperature for 2 h. The membranes were washed and probed using an enhanced chemiluminescence (ECL) kit (Thermo Scientific, Barrington, IL, USA). ImageJ (Version 1.48, National Institute of Health) was used to quantify band density. Antibodies against GFP, C/EBP homologous protein (CHOP), 78-kDa glucose-regulated protein (GRP78), JNK, phospho-JNK, protein kinase R (PKR)-like ER kinase (PERK) and phospho-PERK and GAPDH were purchased from Santa Cruz Biotechnology (Dallas, TX, USA) and were diluted to 1:1000. Antibodies against cleaved poly (ADP-ribose) polymerase (PARP), cleaved caspase-3, IRE1α, apoptosis signal-regulating kinase 1 (ASK1), phospho-ASK1, p38 (a mitogen-activated protein kinase), phospho-p38, X-box binding protein-1 (XBP-1) and TNF receptor-associated factor 2 (TRAF2) were provided by Cell Signaling Technology (Danvers, MA, USA) and were diluted to 1:1000. The antibody to phospho-IRE1 was obtained from Abcam (Cambridge, UK) and was diluted to 1:1000. The antibody to Bax (diluted to 1:1000), and the goat anti-rabbit and goat anti-mouse secondary antibodies (diluted to 1:5000) were purchased from ZSGB-Bio (Beijing, China). Untransfected cells served as a negative control. Cells treated with STS (1 μM, 6 h) and/or TM (1 μM, 24 h) served as positive controls. Cells treated with 4μ8C (100 nM, added 12 h after transfection, for 12 h) and/or SP6000125 (20 μM, added 12 h after transfection, for 12 h) were used to demonstrate the effect of GRA15_II_ on ERS in JEG-3 cells.

### Statistical analysis

A two-tailed independent Student’s t-test (GraphPad Prism 5.0 software, GraphPad Prism, San Diego, CA, USA) was used to determine the differences between control and pEGFP-GRA15_II_-transfected JEG-3 cells. Data are presented as the mean ± standard error (SE). All statistical tests were considered as significant at *P* < 0.05.

## Results

### The presence of GRA15_II_

**Fig. 1:**
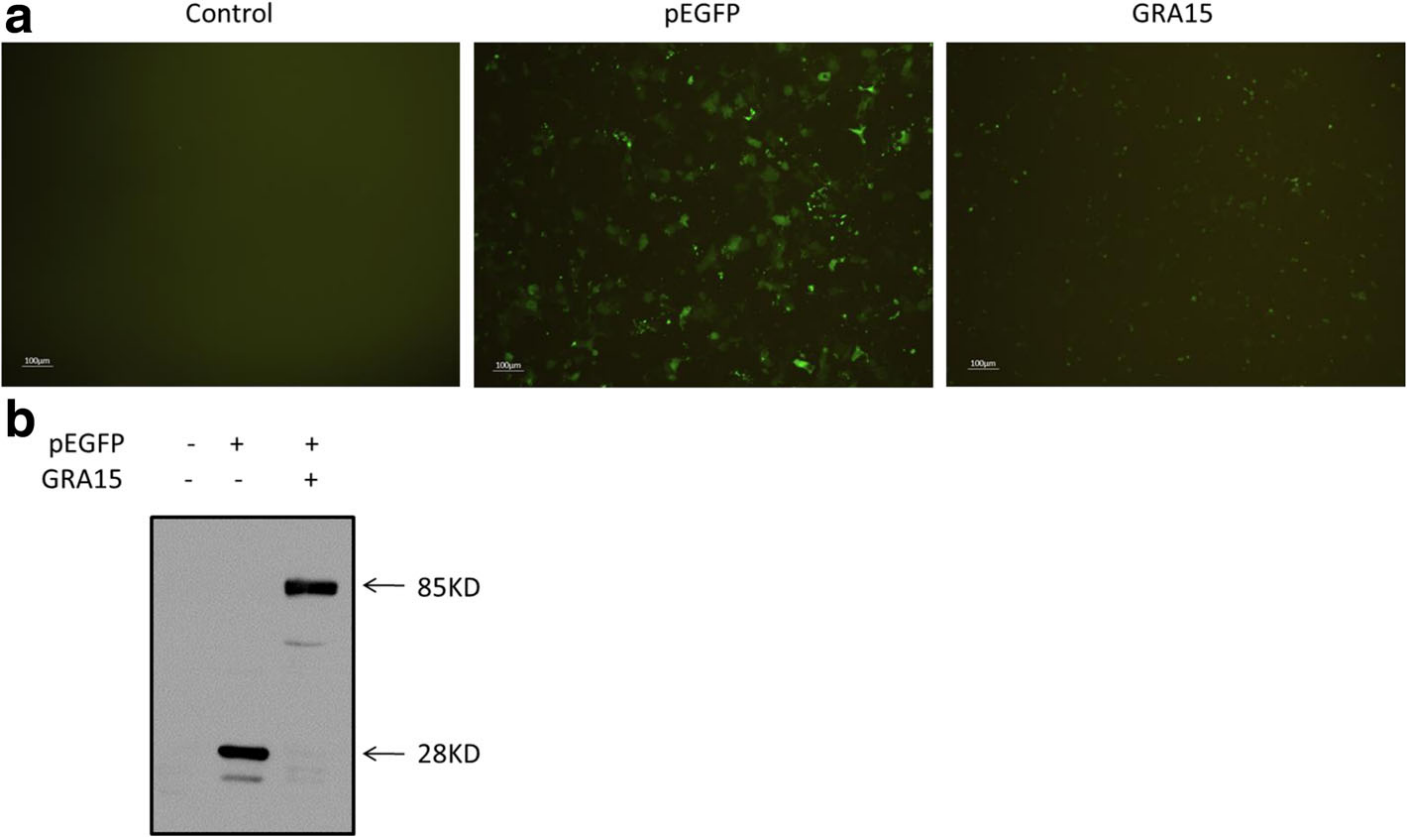
The presence of dense granule protein 15 (GRA15_II_). Choriocarcinoma JEG-3 cells were transfected with either an empty vector (pEGFP, encoding enhanced green fluorescent protein) or pEGFP-GRA15_II_ for 24 h. Untransfected cells served as the control. **a** The expression of green fluorescent protein (GFP) was captured using fluorescent microscopy. *Scale-bar*: 100 μm. **b** The expression of GRA15_II_ was confirmed by western blotting

To investigate whether the pEGFP-GRA15_II_ construct could be expressed in JEG-3 cells, the expression of GRA15_II_ protein was determined in both pEGFP-GRA15_II_- and pEGFP-transfected JEG-3 cells. GFP fluorescence was detected in both pEGFP and pEGFP-GRA15_II_-transfected JEG-3 cells at 24 h (Fig. 1a). pEGFP-transfected cells had an increased fluorescence signal when compared to pEGFP-GRA15_II_-transfected cells. The GFP protein (28 kDa) and the GFP-GRA15_II_ fusion protein (85 kDa) were expressed in JEG-3 cells, 24 h after transfection (Fig. 1b).

### Loss of cell viability and apoptosis

**Fig. 2:**
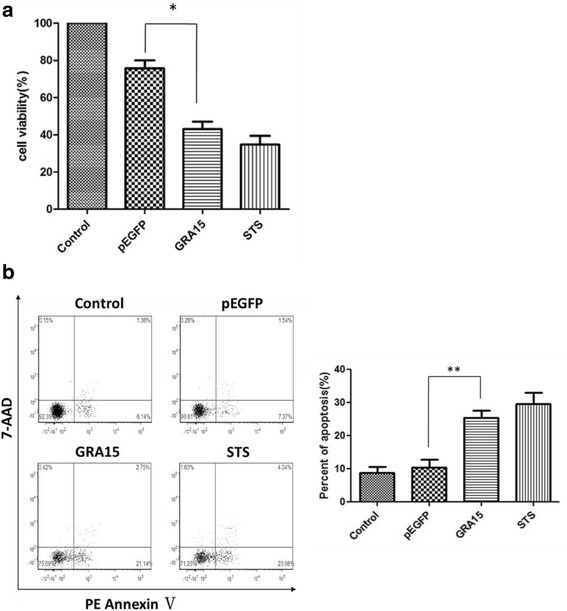
Dense granule protein 15 (GRA15_II_)-induced loss of cell viability and apoptosis. Choriocarcinoma JEG-3 cells were transfected with either empty vector (pEGFP, encoding enhanced green fluorescent protein) or pEGFP-GRA15_II_ for 24 h. Untransfected cells served as the negative control, and staurosporine (STS) treated cells (1 μM, 6 h) served as the positive control. **a** Cell viability was measured using the MTS (3-[4,5-dimethylthiazol-2-yl]-5-[3-carboxymethoxyphenyl]-2-[4-sulfophenyl]-2H-tetrazolium) assay. **b** Cell apoptosis was determined by the phycoerythrin-annexin V/7-AAD flow cytometry assay. *P < 0.05, **P < 0.01

**Fig. 3:**
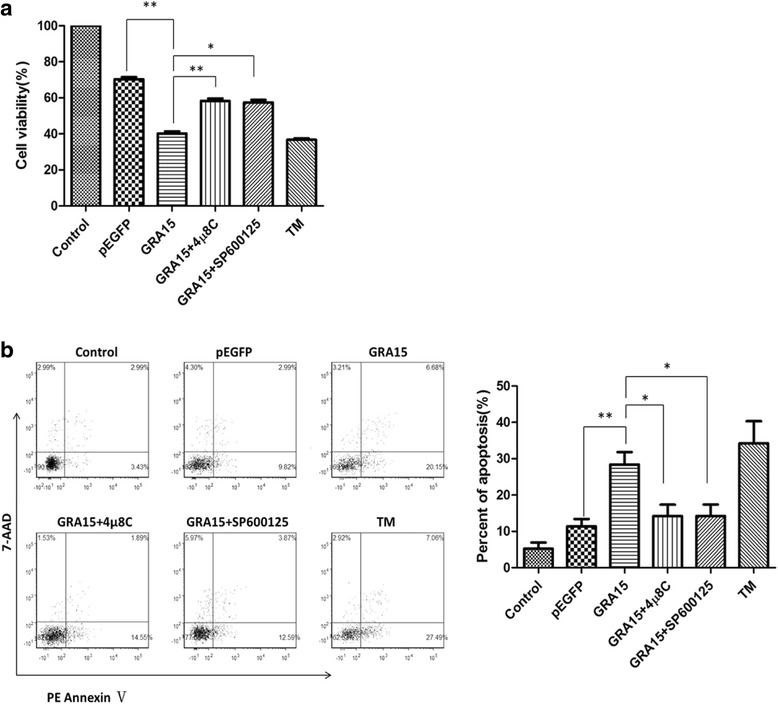
Effects of IRE1α inhibitor 4μ8C and JNK inhibitor SP6000125 on loss of cell viability and apoptosis of pEGFP-GRA15_II_-transfected cells. Choriocarcinoma JEG-3 cells were transfected with either the empty vector (pEGFP, encoding enhanced green fluorescent protein) or pEGFP-GRA15_II_ for 24 h. Tunicamycin (TM) treated (1 μM, 24 h) cells served as the control. Cells were treated with either 4μ8C (100 nM, 12 h) or SP6000125 (20 μM, 12 h) 12 h after pEGFP-GRA15_II_ transfection. **a** Cell viability was measured using the MTS (3-[4,5-dimethylthiazol-2-yl]-5-[3-carboxymethoxyphenyl]-2-[4-sulfophenyl]-2H-tetrazolium) assay. **b** Cell apoptosis was determined by the phycoerythrin-annexin V/7-AAD flow cytometry assay. *P < 0.05, **P < 0.01

Viability and apoptosis, that may directly reflect the functional status of the cells, were analyzed in both pEGFP-GRA15_II_- and pEGFP-transfected JEG-3 cells. Untransfected JEG-3 cells exhibited 100% viability and 7.5% apoptosis (Fig. 2). STS treatment decreased cell viability to 39.4% and increased apoptosis to 27.8%. The pEGFP-GRA15_II_-transfected cells showed decreased cell viability (*t*_(2)_ = 5.611, *P* = 0.0303, 47.0 *vs* 74.5%) and increased cell apoptosis (*t*_(2)_ = 10.74, *P* = 0.0086, 23.9 *vs* 8.9%) when compared to pEGFP-transfected cells. Interestingly, while pEGFP-GRA15_II_ transfection decreased viability (*t*_(2)_ = 10.23, *P* = 0.0094) and increased apoptosis (*t*_(2)_ = 4.851, *P* = 0.04) of JEG-3 cells, treatment with either 4μ8C (IRE1α inhibitor) or SP6000125 (JNK inhibitor) increased viability (*t*_(2)_ = 9,165, *P* = 0.0117) and decreased apoptosis (*t*_(2)_ = 6,963, *P* = 0.02) in pEGFP-GRA15_II_-transfected JEG-3 cells (Fig. 3).

### Transcription levels of apoptosis-associated genes and ERS genes

**Fig. 4:**
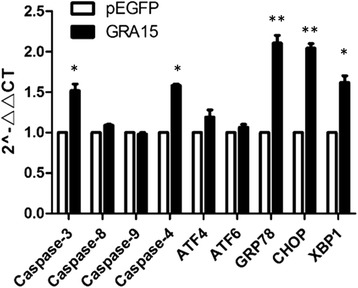
Transcription levels of apoptosis-associated genes. Choriocarcinoma JEG-3 cells were transfected with either the empty vector (pEGFP, encoding enhanced green fluorescent protein) or pEGFP-GRA15_II_ for 24 h. *Abbreviations*: ATF, activating transcription factor; GRP78, 78-kDa glucose-regulated protein; CHOP, C/EBP homologous protein; XBP1, X-box binding protein-1. *P < 0.05, **P < 0.01, when compared to the pEGFP-transfected cells

The transcription levels of apoptosis-associated genes and ERS genes were measured in both pEGFP-GRA15_II_- and pEGFP-transfected JEG-3 cells so as to reveal the signal pathway of the cell apoptosis. The pEGFP-GRA15_II_-transfected JEG-3 cells demonstrated increased mRNA expression levels of caspase-3 (*t*_(2)_ = 6.229, *P* = 0.0248), caspase-4 (*t*_(2)_ = 5.819, *P* = 0.0283), GRP78 (*t*_(2)_ = 11.632, *P* = 0.0073), CHOP (*t*_(2)_ = 18.298, *P* = 0.003) and XBP1 (*t*_(2)_ = 7.589, *P* = 0.0169) when compared to pEGFP-transfected JEG-3 cells (Fig. 4).

### Expression of apoptosis-associated proteins and ERS proteins

**Fig. 5:**
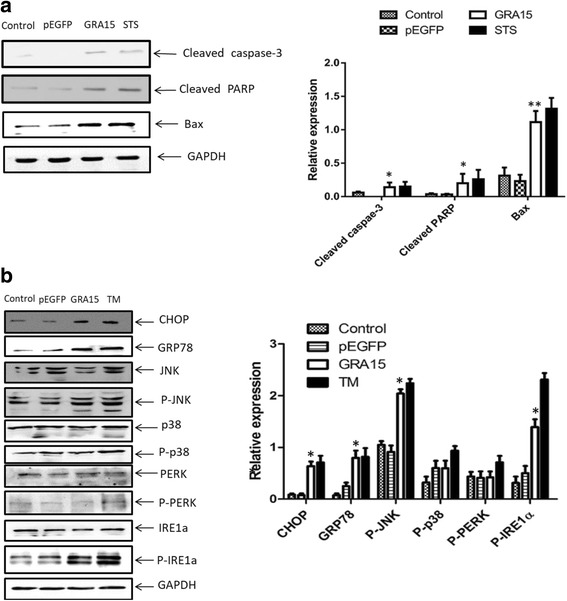
Expression of apoptosis-associated proteins and endoplasmic reticulum stress (ERS) proteins. Choriocarcinoma JEG-3 cells were transfected with either the empty vector (pEGFP, encoding enhanced green fluorescent protein) or pEGFP-GRA15_II_ for 24 h. Untransfected cells served as the negative control. **a** Expression of apoptosis-associated proteins. Staurosporine (STS) treated cells (1 μM, 6 h) served as the positive control. **b** Expression of ERS proteins. Tunicamycin (TM) treated cells (1 μM, 24 h) served as the positive control. *Abbreviations*: PARP, poly (ADP-ribose) polymerase; GAPDH, glyceraldehyde-3-phosphate dehydrogenase; CHOP, C/EBP homologous protein; GRP78, 78-kDa glucose-regulated protein; JNK, c-Jun N-terminal kinase; P-JNK, phosphorylated JNK; P38, a mitogen-activated protein kinase; P-p38, phosphorylated p38; PERK, PRK-like ER kinase; P-PERK, phosphorylated PERK; IRE1α, inositol requiring kinase 1; P-IRE1α, phosphorylated IRE1α. *P < 0.05, **P < 0.01, when compared to the pEGFP-transfected cells

**Fig. 6:**
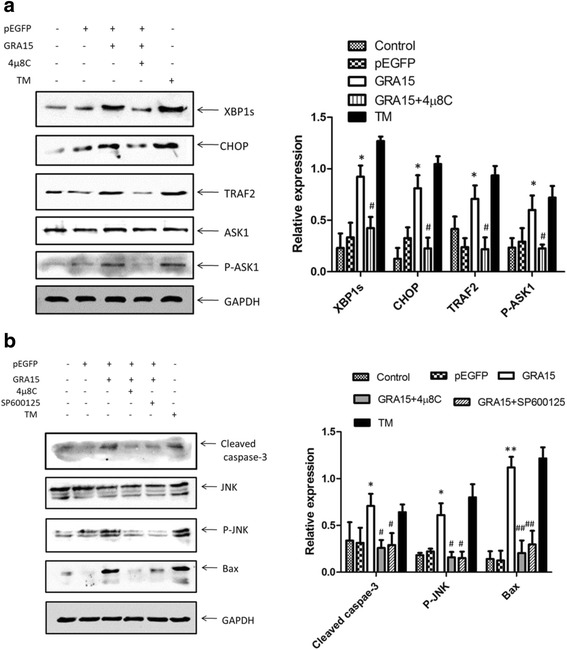
Effects of IRE1α inhibitor 4μ8C and JNK inhibitor SP6000125 on pEGFP-GRA15_II_ transfected cells. Choriocarcinoma JEG-3 cells were transfected with either the empty vector (pEGFP, encoding enhanced green fluorescent protein) or pEGFP-GRA15_II_ for 24 h. Tunicamycin (TM) treated cells (1 μM, 24 h) served as a control. **a** Cells were treated with 4μ8C (100 nM, 12 h) 12 h after pEGFP-GRA15_II_ transfection. *P < 0.05, when compared to pEGFP transfected cells; #*P* < 0.05, when compared with pEGFP-GRA15_II_-transfected cells. **b** Cells were treated with either 4μ8C (100 nM, 12 h) or SP6000125 (20 μM, 12 h) 12 h after pEGFP-GRA15_II_ transfection. *P < 0.05, **P < 0.01, when compared to pEGFP transfected cells; #*P* < 0.05, ##*P* < 0.01, when compared to pEGFP-GRA15_II_-transfected cells. *Abbreviations*: XBP1, X-box binding protein-1; CHOP, C/EBP homologous protein; TRAF2, TNF receptor-associated factor 2; ASK1, apoptosis signal-regulating kinase 1; P-ASK1, phosphorylated ASK1; GAPDH, glyceraldehyde-3-phosphate dehydrogenase; JNK, c-Jun N-terminal kinase; P-JNK, phosphorylated JNK

Accordingly, the expression levels of apoptosis-associated proteins and ERS proteins were measured in both pEGFP-GRA15_II_- and pEGFP-transfected JEG-3 cells. When compared to pEGFP-transfected cells, the pEGFP-GRA15_II_ transfected cells showed increased expression levels of apoptosis-associated proteins (Fig. 5a) such as cleaved caspase-3 (*t*_(2)_ = 4.797, *P* = 0.0408), cleaved PARP (*t*_(2)_ = 4.728, *P* = 0.0419) and Bax (*t*_(2)_ = 24.489, *P* = 0.0017), as well as increased expression levels of ERS proteins (Fig. 5b), such as CHOP, GRP78, phospho-JNK and phospho-IRE1α (all *P* < 0.05). Interestingly, 4μ8C treatment decreased the expression levels of XBP1s, CHOP, TRAF2 and phospho-ASK1 in pEGFP-GRA15_II_-transfected JEG-3 cells (all *P* < 0.05, Fig. 6a). In addition, treatment with either 4μ8C or SP6000125 decreased the expression levels of cleaved caspase-3, phospho-JNK, and Bax in pEGFP-GRA15_II_-transfected JEG-3 cells (all *P* < 0.05, Fig. 6b).

## Discussion

*Toxoplasma gondii* infection can cause abortion, preterm delivery, stillbirth and fetal abnormalities in pregnant animals and humans [30, 31] through three possible mechanisms. First, *Toxoplasma gondii* can be directly transferred to the fetus through the placenta and cause a congenital infection [25]; secondly, maternal physiological and immunological disorders caused by *Toxoplasma gondii* infection can adversely affect fetal development [26]; and thirdly, *Toxoplasma gondii* can induce apoptosis in placental cells [27]. It has been found that the majority of host cells act as bystanders during an acute infection, and apoptosis of these host cells may result from the secretion of certain soluble factors by parasite-infected cells [32, 33]. The composition of the *T. gondii* excreted-secreted antigens (ESAs) is surprisingly complex and only a few microneme proteins, ROPs and GRAs, have been identified [34, 35]. ROP16- and ROP18-induced host cell apoptosis has been previously reported [21, 22]. In this study, GRA15_II_ transfection significantly increased apoptosis and decreased cell viability in choriocarcinoma JEG-3 cells as early as 24 hours post-transfection. These results suggest that GRA15_II_, which exists in *Toxoplasma gondii* strains such as ME49 (type II *Toxoplasma gondii*) and ToxoDB#9 (major *Toxoplasma gondii* strain in China), is a virulence antigen.

**Fig. 7:**
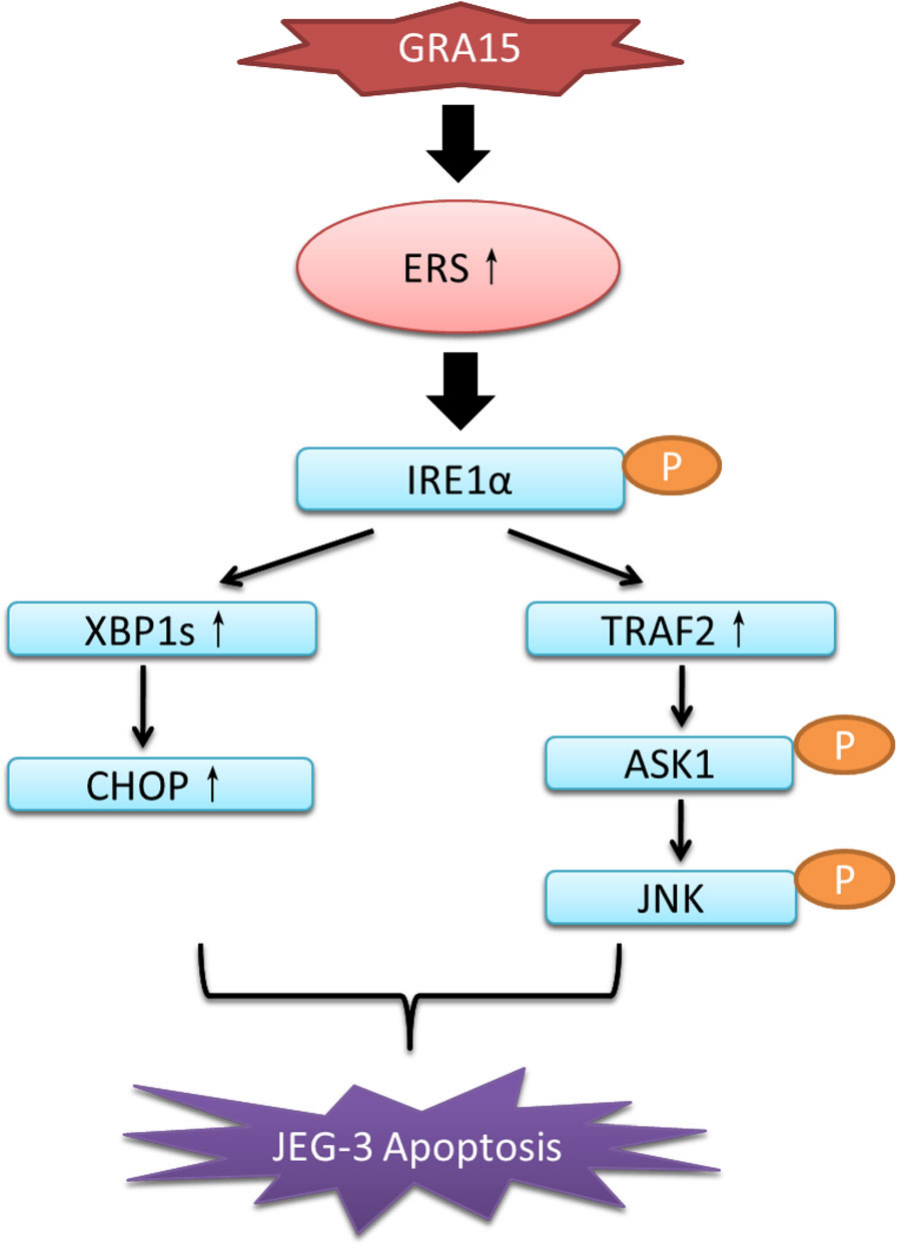
Schematic diagram of the signaling pathways involved in GRA15_II_-induced apoptosis in choriocarcinoma JEG-3 cells. *Abbreviations*: GRA15, dense granule protein 15; ERS, endoplasmic reticulum stress; IRE1α, inositol requiring kinase 1; XBP1, X-box binding protein-1; CHOP, C/EBP homologous protein; TRAF2, TNF receptor-associated factor 2; ASK1, apoptosis signal-regulating kinase 1; JNK, c-Jun N-terminal kinase

The GRA15_II_-induced apoptosis was accompanied with ERS in choriocarcinoma JEG-3 cells. The mRNA transcription and protein expression level of GRP78, a key ERS sensor protein, were significantly increased by GRA15_II_ transfection at 24 hours. The finding indicates that GRA15_II_-induced apoptosis at least partially resulted from ERS. In other studies, PERK, activating transcription factor (ATF) 6, and IRE1α have been proposed as three major proteins downstream of GRP78 signaling during ERS [36, 37]. In the current study, the expression level of phospho-IRE1α, but not PERK or ATF6, was increased by GRA15_II_ transfection. In line with this, the GRA15_II_-related ERS/apoptosis was mainly induced by the GRP78-IRE1α signaling pathway. It has been reported that the activation (phosphorylation) of IRE1α could lead to apoptosis by either mediating the splicing of XBP1 to XBP1s, and subsequently increasing the expression of CHOP [38], or recruiting TRAF2, activating ASK1, and stimulating JNK [39]. In the current work, we found the expression of proteins in both of these pathways was significantly increased by GRA15_II_ transfection. Thus, the result clearly demonstrates that both the IRE1α-XBP1-CHOP and IRElα-TRAF2-ASK1-JNK pathways contributed in GRA15_II_-induced apoptosis as proposed schematically in Fig. 7. We further illustrated this hypothesis by treating the GRA15_II_-transfected choriocarcinoma JEG-3 cells with 4μ8C (IRElα inhibitor) and SP6000125 (JNK inhibitor); both 4μ8C and SP6000125 suppressed the expression levels of apoptosis-associated proteins in GRA15_II_-transfected choriocarcinoma JEG-3 cells, and subsequently decreased apoptosis and increased cell viability.

During pregnancy, fetal development is directly related to the proliferation, differentiation, and apoptosis of trophoblast cells [23, 24]. Increased trophoblast cell apoptosis could be damaging to fetal health and even cause adverse pregnancy outcomes [40, 41]. The above finding contributes novel knowledge to our current understanding in regards to *Toxoplasma gondii*-induced apoptosis, and may help to illustrate the underlying mechanism of *Toxoplasma gondii*-induced pregnancy failure. The objective of the current study was to determine whether ERS was involved in GRA15_II_-induced apoptosis in choriocarcinoma JEG-3 cells. It was not investigated whether ERS was the only (or the major) factor that caused apoptosis in GRA15_II_-transfected choriocarcinoma JEG-3 cells. It is possible that other pathways (e.g. mitochondrial pathway, death receptor pathway) may also contribute to GRA15_II_-induced cell apoptosis, and this will need to be investigated in future studies. Additionally, the host target protein of GRA15_II_ remains unknown. There may be some differences in “normal” *in vivo* cells when compared to the choriocarcinoma JEG-3 cells that were used in the current study.

## Conclusions

*Toxoplasma-*derived GRA15_II_ increased the expression of ERS- and apoptosis-associated proteins in choriocarcinoma JEG-3 cells. GRA15_II_-induced ERS and apoptosis were alleviated by treatment with 4μ8C and SP6000125. GRA15_II_ induces apoptosis at least partially through endoplasmic reticulum stress.

## Abbreviations

GRA15: dense granule protein 15
ROP16: rhoptry protein 16
ERS: endoplasmic reticulum stress
STS: staurosporine
TM: tunicamycin
ATF: activating transcription factor
GRP78: 78-kDa glucose-regulated protein
CHOP: C/EBP homologous protein
XBP1: X-box binding protein-1
PARP: poly (ADP-ribose) polymerase
JNK: c-Jun N-terminal kinase
GAPDH: glyceraldehyde-3-phosphate dehydrogenase
P-JNK: phosphorylated JNK
P38: a mitogen-activated protein kinase
P-p38: phosphorylated p38
PERK: PRK-like ER kinase
P-PERK: phosphorylated PERK
IRE1α: inositol requiring kinase 1
P-IRE1α: phosphorylated IRE1α
TRAF2: TNF receptor-associated factor 2
ASK1: apoptosis signal-regulating kinase 1
P-ASK1: phosphorylated ASK1
